# Dissociative Identity Disorder in Patients Under Stress

**DOI:** 10.1192/j.eurpsy.2025.1957

**Published:** 2025-08-26

**Authors:** N. Kissa, A. Korchi, N. Ait Bensaid, S. Bahetta, A. Ouanass

**Affiliations:** 1 University psychiatric hospital Arrazi; 2 University Psychiatric Hospital Arrazi of Salé Faculty of Medicine and Pharmacy – Mohammed V University of Rabat, Salé, Morocco

## Abstract

**Introduction:**

Dissociative Identity Disorder (DID), as defined in the DSM-5-TR, involves two or more distinct personality states within an individual, along with memory gaps for daily or traumatic events [1]. It is strongly associated with early trauma, such as childhood abuse, and linked to severe psychiatric comorbidities, including depression, anxiety, and self-harm, with an elevated risk of suicide attempts [2][3]. Stress-related neurobiological responses, particularly HPA axis hyperactivity, contribute to identity fragmentation in DID patients. Treatment, often through cognitive-behavioral therapy, aims to integrate dissociated states, reduce self-destructive behaviors, and improve quality of life [2][3].

**Objectives:**

In this case study, we present two compelling cases of dissociative identity disorder following a state of stress. We hope this case study will help reveal the possible association of the disorder with stress

**Methods:**

This is a retrospective and descriptive case study aimed at exploring the manifestations and associated factors of dissociative identity disorder in patients who have experienced traumatic events.

**Results:**

***Clinical Case Summary of Case 1 :***

A 24-year-old student developed dissociative identity disorder after witnessing a fatal accident caused by a drunk driver. This trauma fragmented his personality into three identities: a child expressing needs through crying, a rebellious adolescent who smokes, and a feminine, seductive personality. These identities appear involuntarily, especially during periods of stress, causing him anxiety and memory loss regarding these episodes.

***Clinical Case Summary of Case 2 :***

A 23-year-old single, unemployed patient with a long psychiatric history was admitted following a suicide attempt by jumping. She experiences recurrent depressive episodes, suicidal thoughts, and engages in self-harm to manage her anxiety. Salma has dissociative identity disorder, with an alternate identity named “RUBY” that emerges under stress, driving her towards self-destructive behaviors and exacerbating her distress. Her history includes significant childhood trauma, such as sexual abuse and physical violence, contributing to her identity dissociation. She also exhibits alcohol abuse, consuming up to 1 liter daily to manage her anxiety.

**Conclusions:**

Dissociative identity disorder is a complex condition, often linked to early trauma, characterized by the presence of multiple distinct identities within the same individual. This disorder causes significant distress and impacts patients’ daily functioning, especially in stressful situations. Treatment primarily involves therapeutic approaches aimed at integrating the different identities and reducing self-destructive behaviors. Understanding and managing this disorder require a personalized approach and long-term follow-up.

**Disclosure of Interest:**

None Declared

